# Impact on dose distribution and volume changes of a bioabsorbable polyglycolic acid spacer during chemo-proton therapy for a pediatric Ewing sarcoma

**DOI:** 10.1093/jrr/rraa087

**Published:** 2020-09-22

**Authors:** Mitsuhiro Kimura, Kumiko Asai, Hiromitsu Iwata, Hiroyuki Ogino, Yasuhiko Ito, Michi Kamei, Daisuke Takagi, Naoko Maeda, Yuta Shibamoto

**Affiliations:** Department of Proton Therapy Physics, Nagoya Proton Therapy Center, Nagoya City West Medical Center, Hirate-cho 1-1-1, Kita-ku, Nagoya 462-8508, Japan; Department of Radiology, Nagoya City University Graduate School of Medical Sciences, Kawasumi 1, Mizuho-cho, Mizuho-ku, Nagoya 467-8601, Japan; Department of Proton Therapy Technology, Nagoya Proton Therapy Center, Nagoya City West Medical Center, Hirate-cho 1-1-1, Kita-ku, Nagoya 462-8508, Japan; Department of Radiology, Nagoya City University Graduate School of Medical Sciences, Kawasumi 1, Mizuho-cho, Mizuho-ku, Nagoya 467-8601, Japan; Department of Radiation Oncology, Nagoya Proton Therapy Center, Nagoya City West Medical Center, Hirate-cho 1-1-1, Kita-ku, Nagoya 462-8508, Japan; Department of Radiology, Nagoya City University Graduate School of Medical Sciences, Kawasumi 1, Mizuho-cho, Mizuho-ku, Nagoya 467-8601, Japan; Department of Radiation Oncology, Nagoya Proton Therapy Center, Nagoya City West Medical Center, Hirate-cho 1-1-1, Kita-ku, Nagoya 462-8508, Japan; Department of Pediatric Oncology, Nagoya City West Medical Center, Hirate-cho 1-1-1, Kita-ku, Nagoya 462-8508, Japan; Department of Neonatology and Pediatrics, Nagoya City University Graduate School of Medical Sciences, Kawasumi 1, Mizuho-cho, Mizuho-ku, Nagoya 467-8601, Japan; Department of Oncology, Immunology and Surgery, Nagoya City University Graduate School of Medical Sciences, Kawasumi 1, Mizuho-cho, Mizuho-ku, Nagoya 467-8601, Japan; Department of Pediatrics, National Hospital Organization Nagoya Medical Center, Sannomaru 4-1-1, Naka-ku, Nagoya 460-0001, Japan; Department of Radiology, Nagoya City University Graduate School of Medical Sciences, Kawasumi 1, Mizuho-cho, Mizuho-ku, Nagoya 467-8601, Japan

**Keywords:** polyglycolic acid (PGA) spacer, pediatric cancer, chemo-proton therapy, volume changes

## Abstract

The clinical utility of a recently developed bioabsorbable polyglycolic acid (PGA) spacer has not yet been established in pediatric patients; therefore, we aimed to investigate its utility during chemo-proton therapy for pediatric cancer. Proton depth–dose curves were obtained in a water phantom with or without the spacer. Computed tomography (CT) scans were performed for the PGA spacer immersed in saline for 2 weeks to measure CT numbers and estimate the relative stopping power (RSP) for the proton beams. The spacer was placed in a patient with sacral Ewing sarcoma receiving 55.8 Gy [relative biological effectiveness (RBE)] in 31 fractions and was evaluated using CT scans performed every other week. In addition, the images were used to quantitatively evaluate changes in volume and RSP of the spacer and dose distributions in normal tissues. The spacer immersed in saline had a CT number of 91 ± 7 (mean ± standard deviation) Hounsfield units, and the corresponding RSP was predicted to be 1.07 ± 0.01. The measured RSP agreed with the predicted one. The volumes of the large bowel and rectum receiving ≥45 Gy(RBE) (V_45Gy_) were significantly reduced by placing the spacer; V_45Gy_ without and with the spacer were 48.5 and 0.01%, respectively, for the rectum and 7.2 and 0%, respectively, for the large bowel. The volume of the spacer and RSP decreased at rates of 4.6 and 0.44% per week, respectively, whereas the target dose coverage was maintained until the end of treatment. The PGA spacer was considered effective for pediatric cancer patients undergoing chemo-proton therapy.

## INTRODUCTION

Recent advances in multidisciplinary treatment have improved the survival of pediatric cancer patients, but in exchange, they face the problem of living with late complications [1–4]. Although radiotherapy plays a key role in controlling solid tumors, it is associated with the risk of developing late sequelae [5]. Due to the lower radiation tolerance of normal tissues in children, childhood cancer survivors may have growth disturbance in their bones and late complications in the intestinal tract after radiation therapy, requiring long-term therapeutic intervention. Therefore, extra doses and irradiation volumes in normal organs need to be minimized to prevent long-term adverse effects. Proton therapy is a useful method to reduce radiation damage in pediatric patients, owing to the Bragg peak allowing for sparing normal tissues especially behind the tumor [6–11]. Nevertheless, the dose delivery may sometimes be limited because of adjacent radiosensitive tissues.

Previous studies reported that placement of a spacer GORE-TEX® (W. L. Gore & Associates, Inc., Newark, DE, USA) could effectively displace normal tissues from irradiation fields and provide a dosimetric advantage and long-term clinical benefits throughout the treatment duration [12–14]. However, the GORE-TEX® spacer consists of a non-bioabsorbable material, polytetrafluoroethylene, which remains permanently in the body. Pediatric patients must, therefore, undergo surgery to remove the implanted spacer because it is harmful to the growing bones and intestinal tract. Moreover, chemotherapy has to be suspended during and sometimes after the surgery, leading to a reduction in treatment efficiency.

Recently, a bioabsorbable spacer was developed [15]. This spacer is made of non-woven fabric polyglycolic acid (PGA) with high water absorbency. Any absorbed moisture in the spacer exerts a shielding effect against proton beams. The PGA spacer can maintain its thickness and volume for 3 months prior to degradation by hydrolysis in a living body. Long-term stability and safety of the spacer were confirmed in crab-eating monkeys [15], and its effectiveness has been demonstrated in clinical trials of particle therapy alone in adult patients [16]. However, the PGA spacer has not been shown to be effective in children so far. Infants and children are likely to have electrolyte abnormality and acid–base imbalance during chemotherapy because of higher metabolic turnover of water and lower renal function compared with adults. Moreover, children have a higher body temperature than adults, and temperature and pH are known to affect the degradation rate of PGA. Consequently, it is unknown whether a PGA spacer in children is safe and exhibits similar efficacy to those observed in adults.

The purpose of this study was to physically and clinically investigate the effectiveness of a PGA spacer implanted in a pediatric patient. Two key considerations were: (i) changes in dosimetric parameters on treatment plans and (ii) degradation of the spacer compared with adult patients. Degradation was evaluated through volume and proton stopping power relative to that in water (relative stopping power [RSP]).

## MATERIALS AND METHODS

### PGA in saline

We evaluated tissue equivalence of the PGA spacer for proton irradiation using computed tomography (CT) scans and proton range measurements. A CT image is expressed as a spatial distribution of ‘CT number’, which corresponds to the X-ray linear attenuation coefficient in medium. CT number can be converted into RSP with a heuristic conversion method [17]. The spacer is regarded as tissue equivalent when the RSP obtained from CT numbers agrees with proton RSP [18]. Assuming that changes in the CT number correspond to RSP changes shown by the calibration curve, the proton range and dose distribution can be accurately calculated without overwriting the CT numbers of the spacer.

We prepared a 20 × 10 × 1 cm (thick) PGA spacer (Neskeep®, Alfresa Pharma Co., Osaka, Japan). To simulate the spacer placed in a body, the spacer was soaked in distilled water containing 0.9% NaCl at ~26°C and kneaded enough by hand to remove air. The spacer was CT-scanned (Aquilion One, Canon Medical Systems, Tokyo, Japan) with 2-mm slice thickness at 120-kV voltage, and the images were analyzed with open-source ImageJ software (National Institutes of Health, Bethesda, MD, USA) [19]. A large region of interest was placed in the spacer, excluding the 2-mm thick rims on all sides, and mean CT numbers and their standard deviations (SDs) were obtained automatically. Measured CT numbers were converted to RSP via a calibration curve, which was determined according to the stoichiometric method (see Supplementary Fig. 1) [17].

Proton RSP was determined by calculating the ratio of water equivalent length to physical length of the spacer. The water equivalent length was derived from the shift in the percent depth dose (PDD) curves with and without the spacer. In a water phantom, a parallel plate chamber (Advanced Markus 34045, PTW, Germany) served as a scanning chamber. Measurements were made using passively scattered proton beams delivered from the synchrotron PROBEAT-III (Hitachi Ltd., Tokyo, Japan) at the Nagoya Proton Therapy Center [20, 21]. The spacer containing saline was then irradiated with proton beams with a kinetic energy of 160 MeV with a field size of 25 cm diameter. The range of the proton beams was measured at the 90% distal fall-off with a precision of 0.2 mm. The physical sizes and weights of the spacer were measured using a digital caliper (Mitutoyo, Corp. Kanagawa, Japan) and an electric balance (ER-120A, A&D Company Ltd., Tokyo, Japan), respectively. The physical density was derived from the weight of the spacer divided by its volume.

### PGA spacer in a pediatric patient

CT images and dose distribution in an 11-year-old male patient with Ewing sarcoma at the sacrum were evaluated. The patient received multiagent chemotherapy for tumor size reduction followed by spacer implantation by surgery. A 20 × 10 × 0.5 (thickness) cm PGA spacer was shaped to an appropriate size before implantation. It was folded into a two-layer sandwich and fixed together with an adhesion barrier Seprafilm® (Kaken Pharmaceutical Co. Ltd., Japan) in the retroperitoneal space between the tumor and the intestinal tract. It is encouraged that the spacer be used together with the adhesion barrier to avoid induction of the ileus [16]. There were no acute complications related to the procedure or the device itself.

Following recovery from surgery, the patient received chemotherapy with ifosfamide and etoposide. The patient underwent CT scans with 2-mm slice thickness for treatment planning of proton therapy. Our method for proton therapy planning was described previously [22, 23]. The radiation oncologists delineated the targets and organs at risk (OAR) on the CT images on image registration software MIM Maestro® (MIM software Inc., Cleveland, OH, US). Treatment was planned using the VQA planning system (Hitachi Ltd., Tokyo, Japan) and dose evaluation was made using a relative biological effectiveness (RBE) of 1.1 [21] and the MIM Maestro. The dose constraints were prespecified as follows: the maximum doses (*D*_max_) to the rectum, large bowel, small bowel and bladder ≤55.8 Gy(RBE). The dose covering 98% of a planning target volume (PTV) was planned to be at least 95% of the prescribed dose (D_98%_ > 95%). A single field uniform dose treatment plan of three fields using a spot scanning system was used to deliver a dose of 55.8 Gy(RBE) in 31 fractions to the patient. Proton therapy was performed from the fourth to tenth week after spacer placement.

In order to assess the improvement in dose distribution in the OAR due to the PGA spacer, CT images with 5-mm slice thickness before spacer placement were fused with the treatment planning CT images with bone matching on MIM Maestro using a fusion optimization tool. Then, dummy plans were created and delivered doses on the dummy plans were calculated by overlaying the dose distribution of the treatment plan on the CT images. The impact of the PGA spacer on target coverage and OAR dose was evaluated by comparing dose–volume histograms (DVHs) on the dummy plan and treatment plan.

The patient received verification CT scans at 2-week intervals to check anatomical changes during fractionated therapy. These CT scans provided geometric shapes and RSP of the spacer in the patient’s body. Because the double-layered spacer was expanded by gas produced in the degradation process, we excluded the parts of produced gas when drawing the contours of the spacer manually. The thickness of the spacer could not be measured with high precision because it was inserted along the shape of organs. By using VQA and MIM Maestro, doses at critical organs were checked by applying the beam configurations of the first treatment plan to the anatomy of the verification CT scan. The DVH of the verification plans were compared with the first plan.

## RESULTS

### PGA spacer in saline

**Fig. 1.**
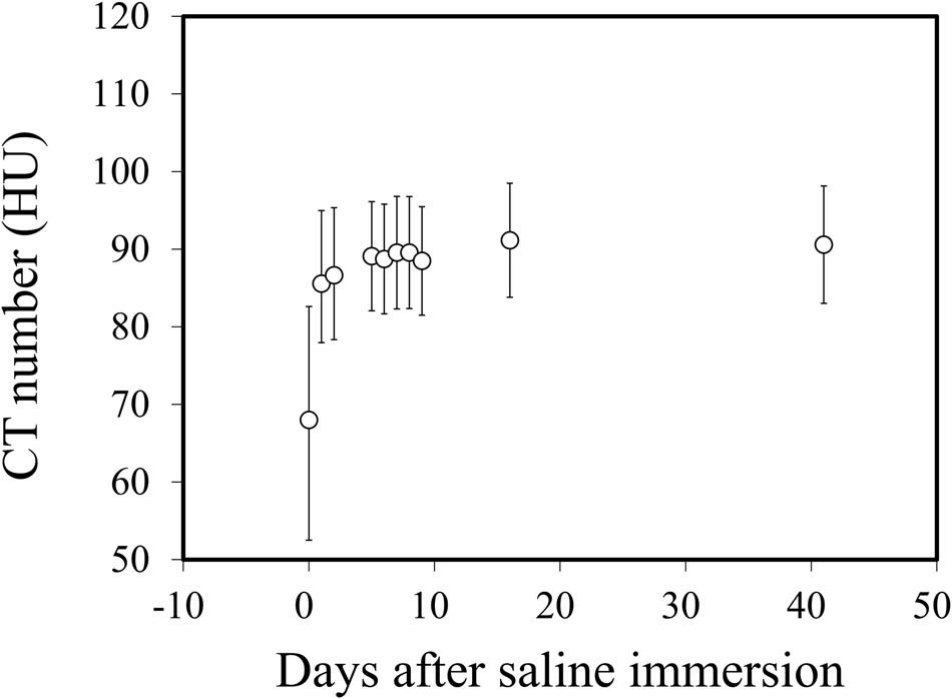
Changes in CT numbers of the PGA spacer as a function of immersion time in saline.

Water absorption of the PGA spacer was measured after it was immersed in saline for 2 weeks. The CT number of the spacer before it was immersed in saline was −730 ± 20 Hounsfield units (HU; mean ± SD) and the CT number of the saline was 18 ± 5 HU. The physical density of the spacer before immersion in saline was 0.22 ± 0.01 g/cm^3^. Figure 1 shows changes in CT numbers of the spacer placed in saline as a function of time after immersion. The CT number of the spacer increased and reached a plateau within 1 week after it was immersed in saline and at 2 weeks it was 91 ± 7 HU; thus, the corresponding RSP was expected to be 1.07 ± 0.01. The physical density of the spacer was 1.06 ± 0.02 g/cm^3^.

The water equivalent shift of the reference Bragg peak position was observed by placing the spacer in the field of the beams. The water equivalent length and physical length of the spacer were measured to be 10.8 ± 0.2 and 10.3 ± 0.1 mm, respectively. The spacer therefore had a proton RSP of 1.06 ± 0.02.

### PGA spacer in a pediatric patient

**Fig. 2.**
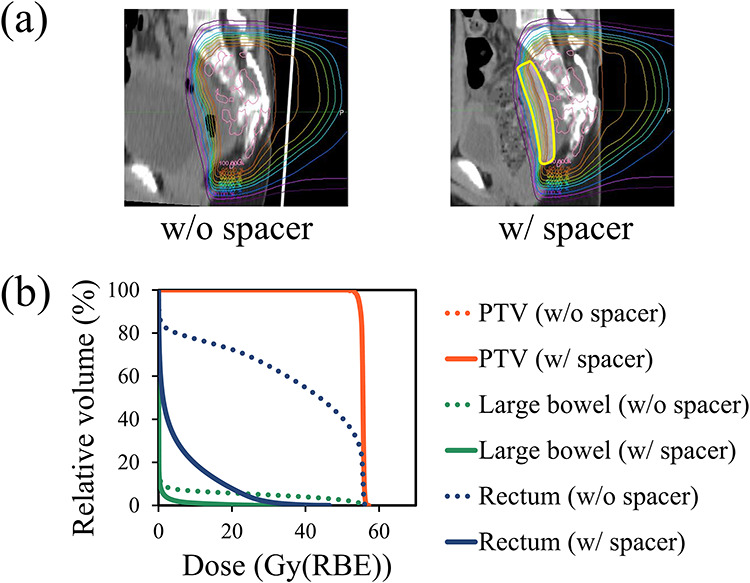
Plan comparison with (w) and without (wo) the PGA spacer in a pediatric patient. (**a**) Plans depicted on axial images at the same anatomical level with and without the PGA spacer. The thick yellow line indicates a contour of the PGA spacer. These dose distributions are normalized using the prescribed dose. (**b**) Comparison of DVHs for the PTV (red), large bowel (green) and rectum (blue) from the treatment plans with and without the spacer.

Figure 2a shows dose distributions with and without the PGA spacer, and Fig. 2b shows corresponding dose–volume histograms. In all these plans, the D_98%_ in the PTV was >95%. On the other hand, the implantation of the PGA spacer resulted in dose reduction in the rectum and large bowel. The D_max_ at the rectum was 56.7 Gy(RBE) before the implantation of the PGA spacer and 46.7 Gy(RBE) after the implantation. Large differences in the volume receiving ≥45 Gy(RBE) (V_45Gy_) in the rectum were found as well: 48.5% before the implantation and 0.01% afterwards. The D_max_ at the large bowel was 56.9 Gy(RBE) before spacer implantation and 34.2 Gy(RBE) after. The V_45Gy_ at the large bowel was 7.2% before and 0% after spacer implantation.

**Fig. 3.**
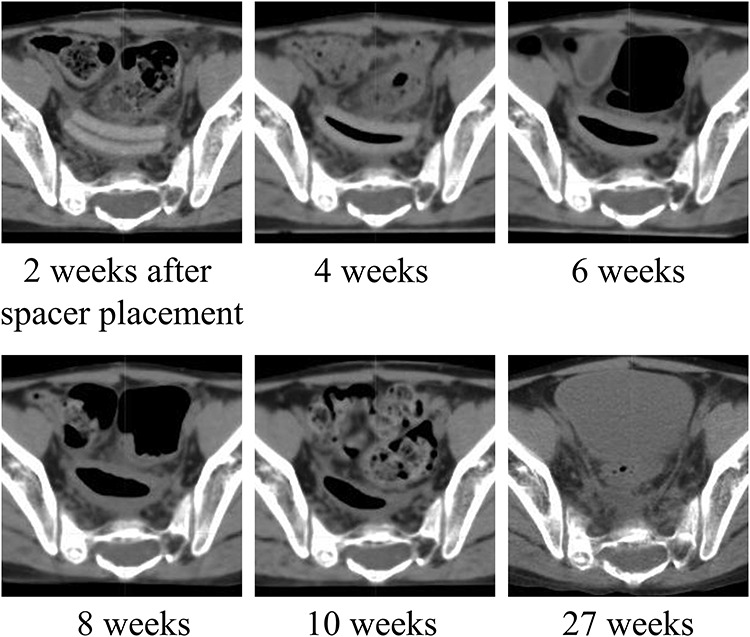
Serial CT images showing morphological changes in spacer reduction over time.

Figure 3 shows changes in CT images of the PGA spacer placed in a pediatric patient over time. CT scans revealed regression of the spacer volume at 6–10 weeks after spacer placement. Air density was observed between the two layers at 4 weeks and thereafter. At 27 weeks, the PGA spacer became invisible.

**Fig. 4.**
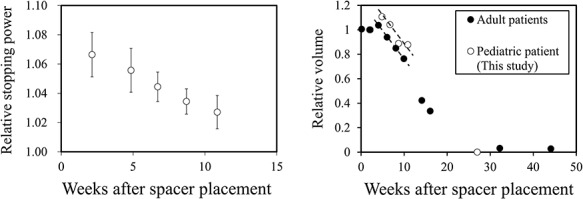
Left panel: changes in the relative stopping power of the PGA spacer placed in a pediatric patient. Right panel: changes in the relative volume of the PGA spacer implanted into adult patients (black circles, plotted from ref. [16]) and the pediatric patient (white circles). The broken lines indicate the volume reduction rate for adult patients.

Figure 4 shows quantitative changes in RSP and relative volume of the PGA spacer placed in the patient as a function of time after surgical placement. The CT number of the spacer after placement in the patient for 2 weeks was 90 ± 23 HU; the corresponding RSP was expected to be 1.07 ± 0.02. The RSP decreased almost linearly at a rate of 0.44% per week. On the other hand, the volume decreased at a rate of 4.6% per week during treatment. Changes in the volume of spacers in adults were plotted for reference from Ref. [16], and the volume reduction rates were similar between adults and our pediatric patient.

**Fig. 5.**
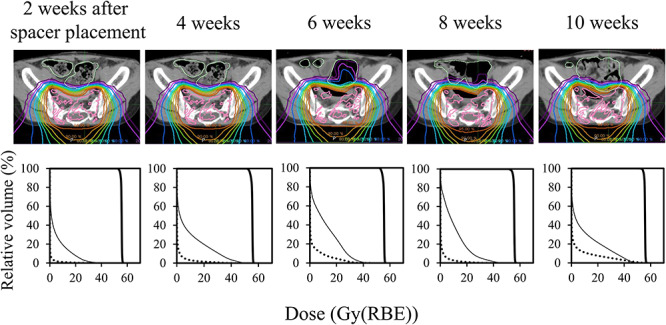
Dose distributions for proton beam therapy (top) and corresponding dose volume histograms (bottom) at different phases, from the development of a treatment plan (left) to the treatment end (right). The dose distributions were normalized using the prescribed dose. The graphs show radiation doses to the planning target volume (thick line), large bowel (dotted line) and rectum (thin line).

Figure 5 shows changes in dose distributions and corresponding DVHs over time, and Table 1 summarizes planning parameters of PTV and OAR for different phases. The spacer placement left a space between the intestinal tract and target tumor and enabled full-dose irradiation to the tumor. The target coverage for the patient was maintained until the end of treatment. The radiation dose given to OAR did not exceed the dose limitations during the treatment; therefore, full-dose irradiation was achieved without modifying the first treatment plan.

**Table 1:** Dosimetric parameters in PTV and OAR at different phases; all values are dose in Gy(RBE)

Organs	Criteria	Elapsed time from spacer placement				
		2 weeks	4 weeks	6 weeks	8 weeks	10 weeks
PTV	D_98%_ > 53.0	53.9	53.1	53.2	53.8	54.1
Rectum	D_max_ ≤ 55.8	46.7	53.1	47.0	49.3	49.3
Large bowel		34.2	43.6	45.6	41.3	55.5
Small bowel		1.1	1.8	1.2	1.4	1.4
Bladder		1.8	3.7	22.7	16.8	2.7

## DISCUSSION

In our phantom study, the mean CT number of the spacer immersed in saline for 2 weeks was 91 HU, and the density of the spacer was almost uniform. This indicted that all parts of the spacer contained sufficient water. The measured RSP of the spacer was in good agreement with the predicted RSP based on CT scan data when the spacer was placed in saline for a sufficient time. These results demonstrated the tissue equivalence of the PGA spacer in wet conditions and suggested that CT number would provide a reliable RSP of the PGA spacer placed in a patient’s body. In our case, the PGA spacer offered a significant dose reduction in the rectum and large bowel, so the patient surely benefited from the use of the spacer.

It is assumed that an increase in the RSP of the PGA spacer resulted from water absorption. The RSPs of the spacers in dry and wet conditions were calculated from their material compositions using a method from Ref. [17] in order to check this assumption. Volume occupancy of the air inside the spacer was estimated to be 86% from the densities of the spacer [16] and PGA [24] in dry conditions. The material composition of the spacer in wet conditions can be reproduced by filling the void inside the spacer with saline. Assuming that the PGA fibers do not degrade in water, the RSPs were expected to be 0.20 and 1.06 in dry and wet conditions, respectively, which were in agreement with our measurements. The results indicated that the difference in the RSP of the PGA spacer was caused by absorbing water.

The sustained effectiveness of a PGA spacer in pediatric patients has been a concern. We did not measure the CT–RSP relationship in a PGA spacer during the degradation process in this study; therefore it is not clear whether the CT number of the degraded spacer provides a reliable RSP. However, we concluded that the tissue equivalence of the degraded spacer might have been ensured because the majority of the spacer is composed of water and PGA molecules are released outward. That is why we calculated dose distributions with CT numbers of the degraded spacer in a patient’s body. Our hypothesis was that the degradation velocity of the PGA spacer implanted in a pediatric patient might be faster than that in adult patients because a pediatric patient body generally tends to induce electrolyte and acid–base imbalance during cancer therapy. CT images revealed regression of the spacer volume probably due to a hydrolysis reaction of the materials in the patient. It was assumed that the reaction generated carbon dioxide gas, which expanded the spacer to form an internal cavity. The morphological change in the spacer may have an influence on the position and volume of OAR, which can also happen when a double-layered spacer is implanted in other patients. We adopted the double-layered structure of a 0.5-cm thick spacer because it was too difficult to trim a 1-cm thick spacer. For subsequent cases, the 1-cm thick spacer has been used to avoid the morphological change. In our clinical case, the spacer showed a volume reduction rate similar to that reported for adults, which was not concordant with our hypothesis. This implied that a PGA spacer placed in a pediatric patient exerted a beneficial effect as seen in adult patients when the patient’s condition is managed well during treatment.

In our case, the RSP reduction rate (0.44% per week) was one order of magnitude lower than the volume reduction rate (4.6% per week). Thus, the RSP change during proton therapy was considered to be of limited significance. Dosimetric analysis showed that the PGA spacer allowed for protecting of OAR throughout the treatment period and suggested that this approach should be effective in pediatric cancer treatment as well as in adults. However, the volume and RSP of the PGA spacer decreases as hydrolysis proceeds, and it becomes difficult to stop proton beams when the treatment is protracted. Its effect has a notable impact on radiotherapy, which requires a longer period especially in pediatric cancer treatment for bone and soft tissue. Future work should therefore include regular CT scans designed to evaluate dose deposition in OAR. In addition, it might be necessary to modify and re-optimize the dose distribution during the course of the patient’s treatment.

It should be noted that this study evaluated the benefit of a PGA spacer applied to only one pediatric patient. So further clinical cases are needed to validate the effectiveness of the PGA spacer. A phase I trial (UMIN000039288) is currently ongoing in order to evaluate the safety and efficacy of the spacer in pediatric patients, and the results are awaited.

## CONCLUSIONS

A bioabsorbable spacer is a promising tool for sparing normal tissues in proton therapy. Our data demonstrate that a spacer implanted into a pediatric patient served the same function as in adult patients. Pediatric patients with bone and soft tissue tumors are often treated with radiation over a longer period than adults, so regular CT scans should be carried out to check the dose distribution in future clinical cases.

## Supplementary Material

Supplementary_Fig1_rraa087
