# Supplementary figures and images for: Impact on dose distribution and volume changes of a bioabsorbable polyglycolic acid spacer during chemo-proton therapy for a pediatric Ewing sarcoma
Source: J Radiat Res. 2020 Sep 22;61(6):952–8. doi: 10.1093/jrr/rraa087 (PMC7674708; doi:10.1093/jrr/rraa087)

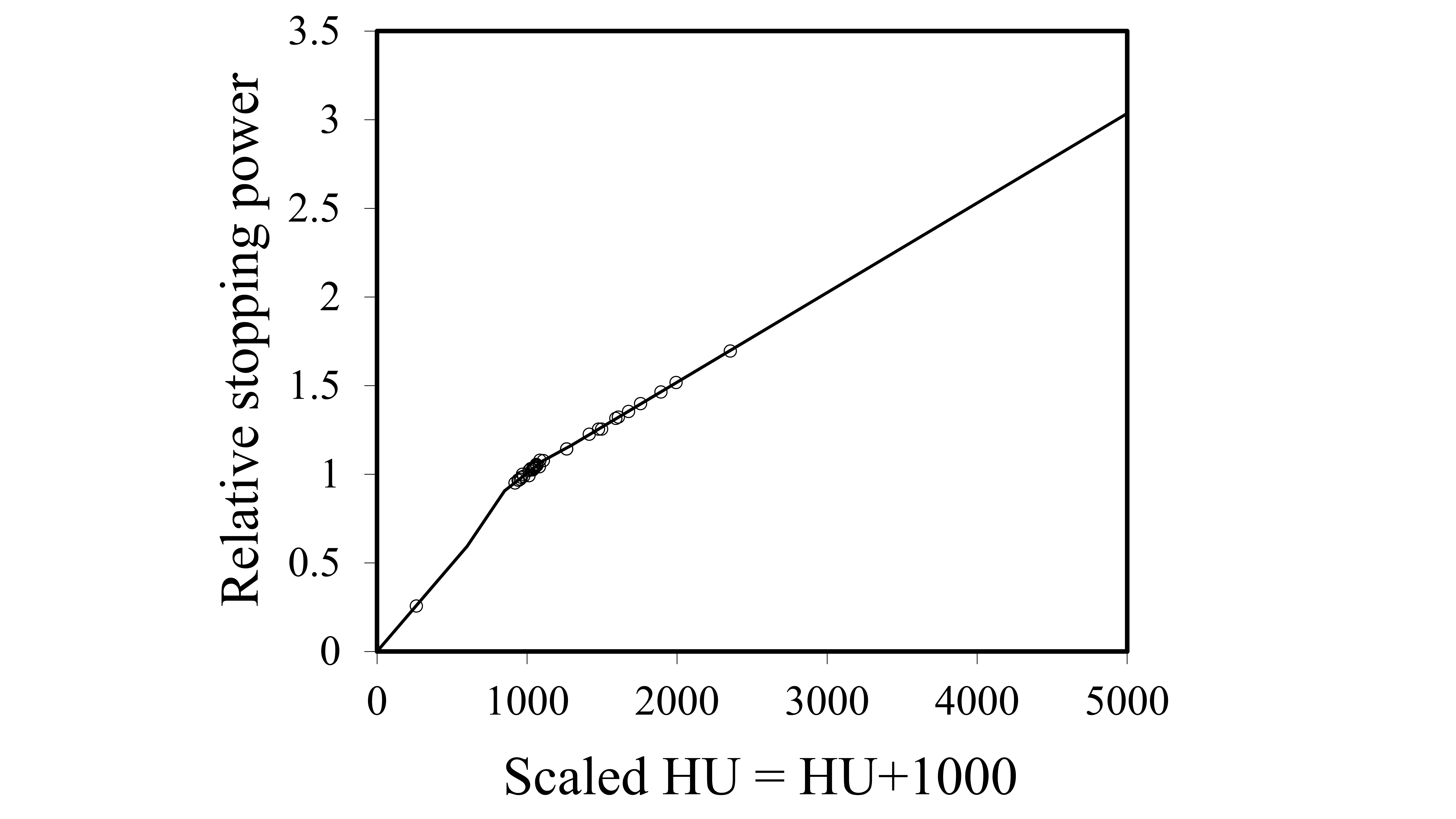

Supplement: Supplementary_Fig1_rraa087 [file supplementary_fig1_rraa087.png]
